# Neural correlates underlying naloxone-induced amelioration of sexual behavior deterioration due to an alarm pheromone

**DOI:** 10.3389/fnins.2015.00052

**Published:** 2015-02-23

**Authors:** Tatsuya Kobayashi, Yasushi Kiyokawa, Yukari Takeuchi, Yuji Mori

**Affiliations:** Laboratory of Veterinary Ethology, Graduate School of Agricultural and Life Sciences, The University of TokyoBunkyo, Tokyo, Japan

**Keywords:** opioid, alarm pheromone, sexual behavior, nucleus paragigantocellularis, periaqueductal gray, paraventricular nucleus of hypothalamus

## Abstract

Sexual behavior is suppressed by various types of stressors. We previously demonstrated that an alarm pheromone released by stressed male Wistar rats is a stressor to other rats, increases the number of mounts needed for ejaculation, and decreases the hit rate (described as the number of intromissions/sum of the mounts and intromissions). This deterioration in sexual behavior was ameliorated by pretreatment with the opioid receptor antagonist naloxone. However, the neural mechanism underlying this remains to be elucidated. Here, we examined Fos expression in 31 brain regions of pheromone-exposed rats and naloxone-pretreated pheromone-exposed rats 60 min after 10 intromissions. As previously reported, the alarm pheromone increased the number of mounts and decreased the hit rate. In addition, Fos expression was increases in the anterior medial division (BNSTam), anterior lateral division (BNSTal) and posterior division (BNSTp) of the bed nucleus of the stria terminalis, parvocellular part of the paraventricular nucleus of the hypothalamus, arcuate nucleus, dorsolateral and ventrolateral periaqueductal gray, and nucleus paragigantocellularis (nPGi). Fos expression was decreased in the magnocellular part of the paraventricular nucleus of the hypothalamus. Pretreatment with naloxone blocked the pheromone-induced changes in Fos expression in the magnocellular part of the paraventricular nucleus of the hypothalamus, ventrolateral periaqueductal gray, and nPGi. Based on these results, we hypothesize that the alarm pheromone deteriorated sexual behavior by activating the ventrolateral periaqueductal gray-nucleus paragigantocellularis cluster and suppressing the magnocellular part of the paraventricular nucleus of the hypothalamus (PVN) via the opioidergic pathway.

## Introduction

Sexual behavior is very important for reproduction in animals. It can be suppressed by various types of manipulations; for example, social isolation (Barrot et al., 2005), maternal separation (Rhees et al., 2001), prenatal stress (Wang et al., 2006), parasitic infection (Lin et al., 1990; Klein, 2003), immobilization, foot shock, and water immersion (Retana-Marquez et al., 1996) suppress male sexual behavior in rodents. In addition, we have found that an alarm pheromone released from stressed conspecifics deteriorates male sexual behavior in rats (Kobayashi et al., 2011).

It is well-known that stressed animals release specific odors, possibly to warn or alarm nearby conspecifics, some of which are defined as alarm pheromones (Karlson and Luscher, 1959; Inagaki et al., 2014). We previously investigated an alarm pheromone that causes an increase in body temperature in rats placed in a box, which recently placed two male rats that had received foot shocks (Kikusui et al., 2001). This phenomenon suggests that the rats that received the foot shocks served as pheromone donors and released an alarm pheromone when they received the shocks. It is possible to induce pheromone release by applying electrical stimulation to the perianal region of an anesthetized donor (Kiyokawa et al., 2004); therefore, we established a method that traps this pheromone in water (Kiyokawa et al., 2005a). We found that this pheromone-containing water can evoke a variety of stress responses, depending on the experimental model (Kiyokawa et al., 2006; Inagaki et al., 2008; Kobayashi et al., 2011), through the vomeronasal system (Kiyokawa et al., 2007, 2013a) and main olfactory system (Inagaki et al., 2014).

This alarm pheromone is a suitable candidate to analyze the effects of stress on sexual behavior because the natural stress responses were induced through the olfactory system. We observed the sexual behavior of pairs of rats in the presence of this pheromone and found that it increases the number of mounts and decreases the hit rate in male rats (Kobayashi et al., 2011). Subsequently, we found that this pheromone-induced deterioration in sexual behavior is gender-specific because it occurs when the pheromone was presented to the male, but not the female, before a sexual encounter (Kobayashi et al., 2011). We conducted Fos mapping in a variety of brain regions and found that the nucleus paragigantocellularis (nPGi) is a key region associated with the pheromone-induced deterioration. We have found increased Fos expression in the anterior medial division (BNSTam), anterior lateral division (BNSTal), and posterior division (BNSTp) of the bed nucleus of the stria terminalis, medial and basal amygdala, and corticotropin-releasing hormone (CRH)-containing neurons in the paraventricular nucleus of the hypothalamus (PVN), which are in the parvocellular part of the PVN (pPVN) (Kobayashi et al., 2013a). However, we did not find any other candidate nuclei that were directly involved in this deterioration or that activated the nPGi. This failure might be attributed to neural activation from ejaculation, which obscured Fos expression associated with pheromone-induced sexual behavior deterioration.

In parallel with the immunohistochemical analyses, we pharmacologically analyzed the neural mechanism of deterioration. We found that systemic pretreatment with the opioid receptor antagonist naloxone attenuated sexual deterioration in male rats. This suggests that opioids are involved in the neural mechanism (Kobayashi et al., 2013b). However, regions in which we found increased Fos expression during deterioration (Kobayashi et al., 2013a), such as the nPGi and several other nuclei, to our knowledge, do not express opioid receptors. Therefore, it remains unclear how opioids are involved in the deterioration of sexual behavior by the alarm pheromone.

To address these issues, in this study, we measured Fos expression in 31 brain regions in pheromone-exposed rats that showed a deterioration of sexual behavior but did not achieve ejaculation. We also assessed pheromone-exposed rats that were pretreated with naloxone in order to obtain further information about the role of opioids in deterioration.

## Material and methods

### Animals

Twenty-five sexually naïve male (aged 7.5 weeks) and female (aged 8.5 weeks) Wistar Imamichi rats were used in this study (Institute for Animal Reproduction, Ibaraki, Japan). All animals were provided with food and water *ad libitum* and kept on a 12-h light-dark cycle (lights turned off at 2000). The colony room was maintained at a constant temperature (24 ± 1°C) and humidity (40–45%). Animals of the same sex were housed in pairs in wire-topped, transparent cages (410 × 250 × 180 mm) with wood shavings for bedding. All procedures were approved by the Animal Care and Use Committee of the Faculty of Agriculture of The University of Tokyo.

### Preparation of the alarm pheromone

Before the sexual behavior test, water samples that contained either the alarm pheromone or a control odor were prepared according to a previously described method (Kiyokawa et al., 2005a; Kobayashi et al., 2013b). Adult male Wistar Imamichi rats (aged 12–16 weeks) were anesthetized and 2 intradermal needles (27G) were attached at either the neck or perianal region. After spraying purified water (5 mL) on the ceiling of an acrylic box (200 × 200 × 100 mm), the anesthetized donor was placed in the box and given 15 electrical stimuli (10 V for 1 s) through the needles for 5 min at 20 s intervals. Care was taken that water droplets did not fall from the ceiling and that the donor was kept under anesthesia during the stimulations. The electrical stimulation of the perianal region induced the release of alarm pheromones and stimulation of the neck region was conducted to provide a similar amount of control olfactory stimuli. Following this, the donor was removed and the water droplets containing the alarm pheromone or control odor were collected. The samples were stored at 4°C until use.

### Sexual behavior test

Sexual behavior tests were conducted as described in previous studies (Kobayashi et al., 2011, 2013a, b). Briefly, 1 day before the test, male rats were housed individually and acclimatized for 30 min to the experimental room and devices. Female rats were also acclimatized to the experimental room for 30 min. On the test day, the test was conducted in the home cage of the male subject that had been placed in the experimental room under a dim red light. Saline or naloxone (40 mg·kg^−1^ dissolved in saline; Sigma-Aldrich, St. Louis, MO) was administered intraperitoneally to the male 60 min before the test. At the beginning of the test, pheromone or control samples (750 μL each) were dropped on two sheets of filter paper (5 × 5 cm) that were placed in acrylic plates (12 × 6 cm, 3 mm thickness), one of which had 18 holes, 9 mm in diameter. The plates were then placed in the home cage for 5 min. Following this, the plates were removed and the female was introduced. Sexual behavior was video recorded and observed in an adjacent room during the test. When the tenth intromission was observed, the female was removed. Twenty-five male rats were divided into three groups according to the type of sample and injection: control odor and saline injection (Control-SAL; *n* = 8), alarm pheromone and saline injection (Pheromone-SAL; *n* = 8), and alarm pheromone and naloxone injection (Pheromone-NAL; *n* = 9).

### Immunohistochemistry

Immunohistochemistry was conducted as described in previous studies (Kiyokawa et al., 2013b, 2014; Kobayashi et al., 2013a). Briefly, 60 min after the tenth intromission, male rats were deeply anesthetized with sodium pentobarbital and perfused intracardially with 0.9% saline followed by 4% paraformaldehyde in 0.1 M phosphate buffer. The brain was removed and immersed overnight in the same fixative and then placed in 30% sucrose in phosphate buffer for cryoprotection. The brain was cut into 25-μm-thick coronal sections. Six successive sections containing some of the target regions were collected, and the second and fifth sections were stained with Cresyl violet to confirm localization in the brain. The remaining sections were incubated in H_2_O_2_, then primary antibody targeting the Fos protein (PC38, Merck Millipore, Billerica, MA) for 65 h, and biotinylated anti-rabbit secondary antibody (BA-1000, Vector Laboratories, Burlingame, CA) for 2 h. Following this, the sections were processed using the ABC kit (Vector Laboratories) and developed using a 3,3′-diaminobenzidine solution with nickel intensification.

### Quantification

Fos expression was analyzed as described in previous studies (Kiyokawa et al., 2005b, 2013b; Kobayashi et al., 2013a). Four sections showing each of the 31 regions were imaged using a microscope equipped with a digital camera (DP30BW; Olympus, Tokyo, Japan). The number of c-Fos-immunoreactive cells in a 0.5-mm square was counted bilaterally using Image J software (version 1.41) by an experimenter who was blind to the experimental groups. The raphe nuclei are located in the center of the brain; therefore, Fos expression could not be counted bilaterally. In some cases, the designated area to be counted was smaller than the boundaries of the 0.5-mm square; therefore, only the cells in the brain region of interest, and not in other regions within the square, were counted.

### Data analysis and statistical procedures

Male sexual behavior was analyzed as described in previous studies by a researcher who was blind to the experimental conditions (Kobayashi et al., 2011, 2013a, b). The following measures of sexual behavior were recorded: mount latency (pelvic thrusting from the rear of the female rat without penile insertion), intromission latency (deeper pelvic thrusting from the rear of the female rat with penile insertion), latency for tenth intromission (time from the first intromission to tenth intromission), and number of mounts (number of mounts needed for 10 intromissions). In addition, the hit rate was calculated. Behavioral and immunohistochemical data are expressed as mean ± the standard error of the mean (SEM). The significance was set at *P* < 0.05 for all statistical tests. All data were analyzed by one-way analysis of variance followed by Fisher's PLSD *post-hoc* test.

## Results

**Figure 1:**
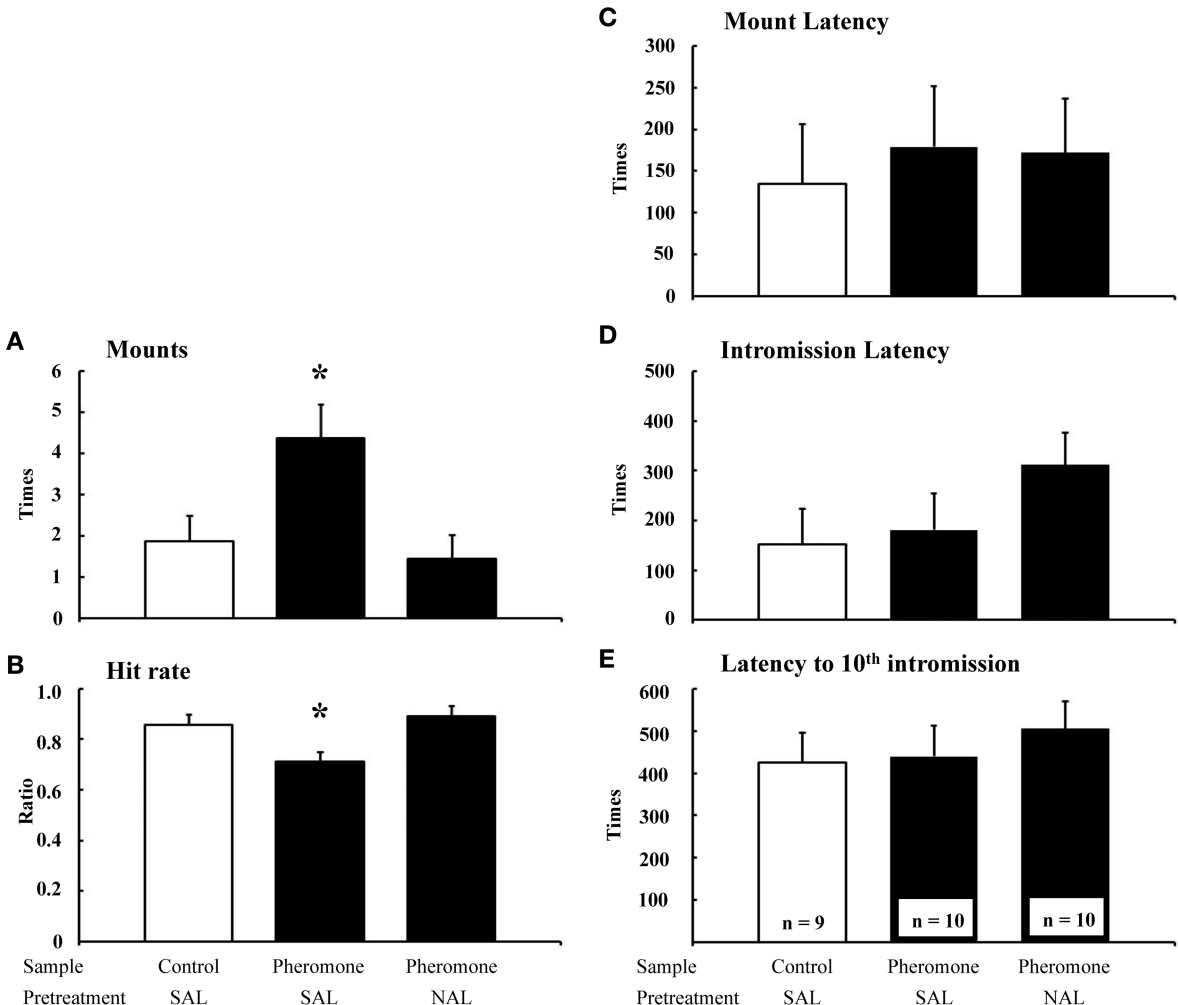
**The effects of naloxone on the pheromone-induced deterioration of sexual behavior**. Number of mounts **(A)**, hit rate **(B)**, mount latency **(C)**, intromission latency **(D)**, and latency to 10th intromission **(E)** of the subjects (mean + SEM). Subjects were pretreated with either saline (SAL) or naloxone (40 mg·kg^−1^; NAL) and exposed to a control odor (Control) or an alarm pheromone (Pheromone). ^*^P < 0.05 compared with the Control-SAL group, ANOVA followed by Fisher's PLSD *post-hoc* test.

The number of mounts [*F*_(2, 22)_ = 5.47, *P* = 0.012; Figure 1A] and hit rate [*F*_(2, 22)_ = 5.40, *P* = 0.012; Figure 1B] were significantly different between the groups, whereas the mount latency [*F*_(2, 22)_ = 0.11, *P* = 0.892; Figure 1C], intromission latency [*F*_(2, 22)_ = 0.68, *P* = 0.519; Figure 1D], and latency to tenth intromission [*F*_(2, 22)_ = 0.30, *P* = 0.746; Figure 1E] were not. *Post-hoc* tests revealed that the Pheromone-SAL group showed an increased number of mounts (*P* = 0.017) and decreased hit rate (*P* = 0.022) when compared with the Control-SAL group. However, these measures were not different between the Control-SAL and Pheromone-NAL groups (number of mounts, *P* = 0.653; hit rate, *P* = 0.565). A *post-hoc* test also confirmed that the Pheromone-SAL group showed an increased number of mounts (*P* = 0.005) and decreased hit rate (*P* = 0.005) when compared with the Pheromone-NAL group.

**Figure 2:**
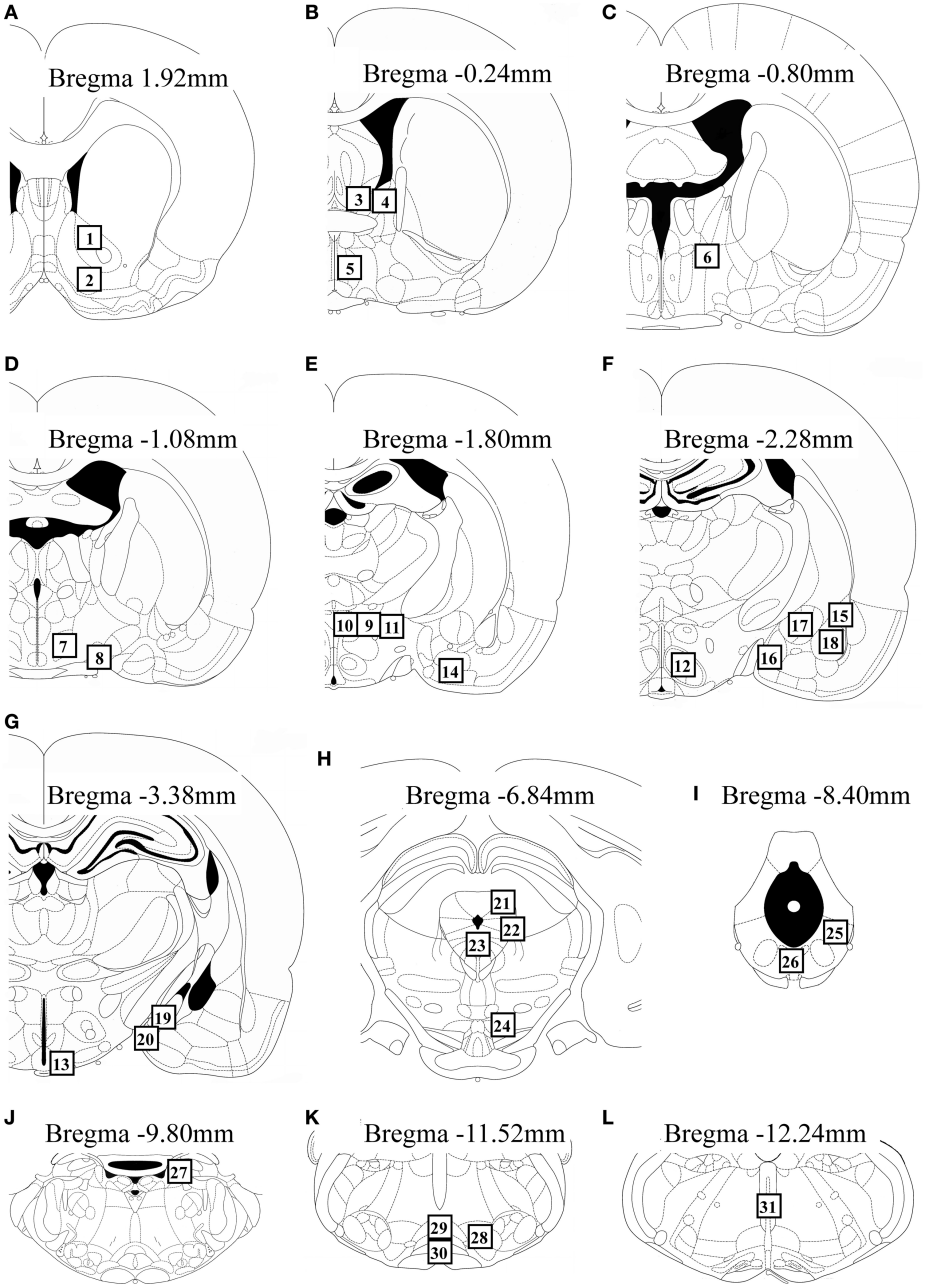
**Schematic diagrams showing the location of brain regions (open square containing numbers) in which c-Fos-immunoreactive cells were counted**. The regions analyzed were: the nucleus accumbens core and shell **(A)**, BNSTam, BNSTal, and medial preoptic area **(B)**, BNSTp **(C)**, lateral hypothalamic area and supraoptic nucleus **(D)**, mPVN, pPVN, peduncular lateral hypothalamus, and anterior cortical amygdala **(E)**, ventromedial nucleus of hypothalamus and lateral, anterior medial, central, and basolateral amygdala **(F)**, Arc and posterodorsal and posteroventral medial amygdala **(G)**, dlPAG, lateral periaqueductal gray, dorsal raphe nucleus, and ventral tegmental area **(H)**, vlPAG and dorsal region of the dorsal raphe nucleus **(I)**, locus coeruleus **(J)**, nPGi and raphe magnus and pallidus **(K)**, and raphe obscurus **(L)**. This figure was adapted from a rat brain atlas (Paxinos and Watson, 2007). For an explanation of the abbreviations, see the corresponding numbers in Table 1.

We measured Fos expression in 31 brain regions (Figure 2). The mean number of c-Fos-immunoreactive cells is summarized in Table 1. Fos expression was significantly different among the groups in the nucleus accumbens core [*F*_(2, 22)_ = 8.21, *P* = 0.002] and shell [*F*_(2, 22)_ = 6.07, *P* = 0.008], BNSTam [*F*_(2, 22)_ = 4.81, *P* = 0.019], BNSTal [*F*_(2, 22)_ = 12.9, *P* < 0.001], BNSTp [*F*_(2, 22)_ = 6.66, *P* = 0.006], magnocellular part of the PVN [mPVN; *F*_(2, 22)_ = 8.09, *P* = 0.002], pPVN [*F*_(2, 22)_ = 8.28, *P* = 0.002], ventromedial nucleus of hypothalamus [*F*_(2, 22)_ = 5.55, *P* = 0.011], arcuate nucleus [Arc; *F*(2, 22) = 12.3, *P* < 0.001], central amygdala [*F*_(2, 22)_ = 16.0, *P* < 0.001], dorsolateral [dlPAG; *F*_(2, 22)_ = 5.53, *P* = 0.011] and ventrolateral periaqueductal gray [vlPAG; *F*_(2, 22)_ = 28.0, *P* < 0.001], dorsal raphe nucleus [*F*_(2, 22)_ = 6.11, *P* = 0.008], and nPGi [*F*_(2, 22)_ = 21.9, *P* < 0.001].

**Table 1:** **The number of c-Fos-immunoreactive cells per 0.25 mm^2^ in various brain regions**.

**Regions**	**Control-SAL (8)**	**Pheromone-SAL (8)**	**Pheromone-NAL (9)**
**THE REGIONS WHERE PRETREATMENT WITH NALOXONE BLOCKED THE CHANGE BY THE PHEROMONE**			
9. Magnocellular part of the paraventricular nucleus of hypothalamus (mPVN)	17.4 ± 2.0	9.8 ± 1.0^*^ *P* = 0.003	17.9 ± 1.6 *P* = 0.795
25. Ventrolateral periaqueductal gray (vlPAG)	33.2 ± 2.8	71.9 ± 6.7^*^ *P* < 0.001	32.9 ± 2.1 *P* = 0.969
28. Nucleus paragigantocellularis (nPGi)	7.3 ± 0.8	21.5 ± 2.6^*^ *P* < 0.001	5.7 ± 0.7 *P* = 0.226
**THE REGIONS WHERE PRETREATMENT WITH NALOXONE DID NOT BLOCK THE ACTIVATION BY THE PHEROMONE**			
3. Anterior medial division of the bed nucleus of the stria terminalis (BNSTam)	18.0 ± 1.4	33.2 ± 4.3^*^ *P* = 0.009	30.9 ± 4.3^*^ *P* = 0.020
4. Anterior lateral division of the bed nucleus of the stria terminalis (BNSTal)	16.5 ± 4.5	34.0 ± 4.0^*^ *P* = 0.012	48.1 ± 4.7^*^ *P* < 0.001
6. Posterior division of the bed nucleus of the stria terminalis (BNSTp)	19.9 ± 2.3	37.7 ± 5.9^*^ *P* = 0.004	36.3 ± 2.3^*^ *P* = 0.005
10. Parvocellular part of the paraventricular nucleus of hypothalamus (pPVN)	34.6 ± 3.0	51.6 ± 4.8^*^ *P* = 0.036	64.7 ± 6.7^*^ *P* < 0.001
13. Arcuate nucleus (Arc)	9.4 ± 1.9	30.3 ± 4.7^*^ *P* = 0.001	34.6 ± 4.1^*^ *P* < 0.001
21. Dorsolateral periaqueductal gray (dlPAG)	16.2 ± 1.6	23.6 ± 1.8^*^ *P* = 0.007	22.9 ± 1.7^*^ *P* = 0.011
**THE REGIONS WHERE PRETREATMENT WITH NALOXONE, BUT NOT PHEROMONE EXPOSURE, CAUSED ACTIVATION**			
1. Nucleus accumbens core	31.5 ± 6.7	39.0 ± 7.6 *P* = 0.470	69.2 ± 6.9^*^ *P* = 0.001
2. Nucleus accumbens shell	7.4 ± 2.4	5.0 ± 1.4 *P* = 0.436	15.0 ± 2.4^*^ *P* = 0.020
12. Ventromedial nucleus of hypothalamus	8.0 ± 1.0	9.2 ± 1.4 *P* = 0.568	14.4 ± 1.8^*^ *P* = 0.005
17. Central amygdala	8.7 ± 1.3	7.6 ± 2.2 *P* = 0.939	74.9 ± 15.5^*^ *P* < 0.001
23. Dorsal raphe nucleus	6.0 ± 0.8	6.3 ± 1.1 *P* = 0.813	10.0 ± 0.8^*^ *P* = 0.005
**THE REGIONS WHERE NEITHER THE PHEROMONE NOR PRETREATMENT WITH NALOXONE CAUSED ACTIVATION**			
5. Medial preoptic area	81.9 ± 7.7	90.6 ± 6.2	88.8 ± 3.9
7. Lateral hypothalamic area	13.9 ± 2.9	17.8 ± 2.5	19.6 ± 2.8
8. Supraoptic nucleus	13.8 ± 3.2	13.5 ± 2.6	8.9 ± 0.9
11. Peduncular lateral hypothalamus	16.1 ± 3.1	20.2 ± 2.4	25.6 ± 2.9
14. Anterior cortical amygdala	36.5 ± 4.1	34.6 ± 5.2	34.2 ± 4.0
15. Lateral amygdala	6.5 ± 1.0	6.8 ± 1.1	7.7 ± 0.5
16. Anterior medial amygdala	24.0 ± 3.1	32.4 ± 3.3	31.9 ± 2.8
18. Basal amygdala	13.6 ± 2.0	12.3 ± 2.6	14.0 ± 2.3
19. Posterodorsal medial amygdala	39.0 ± 3.8	42.7 ± 2.1	40.1 ± 3.0
20. Posteroventral medial amygdala	28.5 ± 5.8	45.6 ± 5.4	44.0 ± 5.9
22. Lateral periaqueductal gray	16.8 ± 2.0	21.9 ± 1.6	25.8 ± 3.5
24. Ventral tegmental area	4.2 ± 0.8	4.1 ± 0.9	4.4 ± 0.7
26. Dorsal raphe nucleus dorsal part	11.7 ± 1.8	11.2 ± 2.0	13.3 ± 1.6
27. Locus coeruleus	13.3 ± 2.5	20.0 ± 3.1	15.8 ± 2.6
29. Raphe magnus	6.0 ± 0.8	7.0 ± 1.6	6.4 ± 1.2
30. Raphe pallidus	4.9 ± 0.5	5.0 ± 0.4	5.5 ± 1.0
31. Raphe obscurus	11.9 ± 2.7	12.2 ± 2.8	18.1 ± 2.1

Data are expressed as means ± SEM. For the location of each region, see the corresponding numbers in Figure 2. The number of rats per group is given in parentheses.

* P < 0.05 compared to the Control-SAL group, One-Way ANOVA followed by Fisher's PLSD post-hoc test.

**Figure 3:**
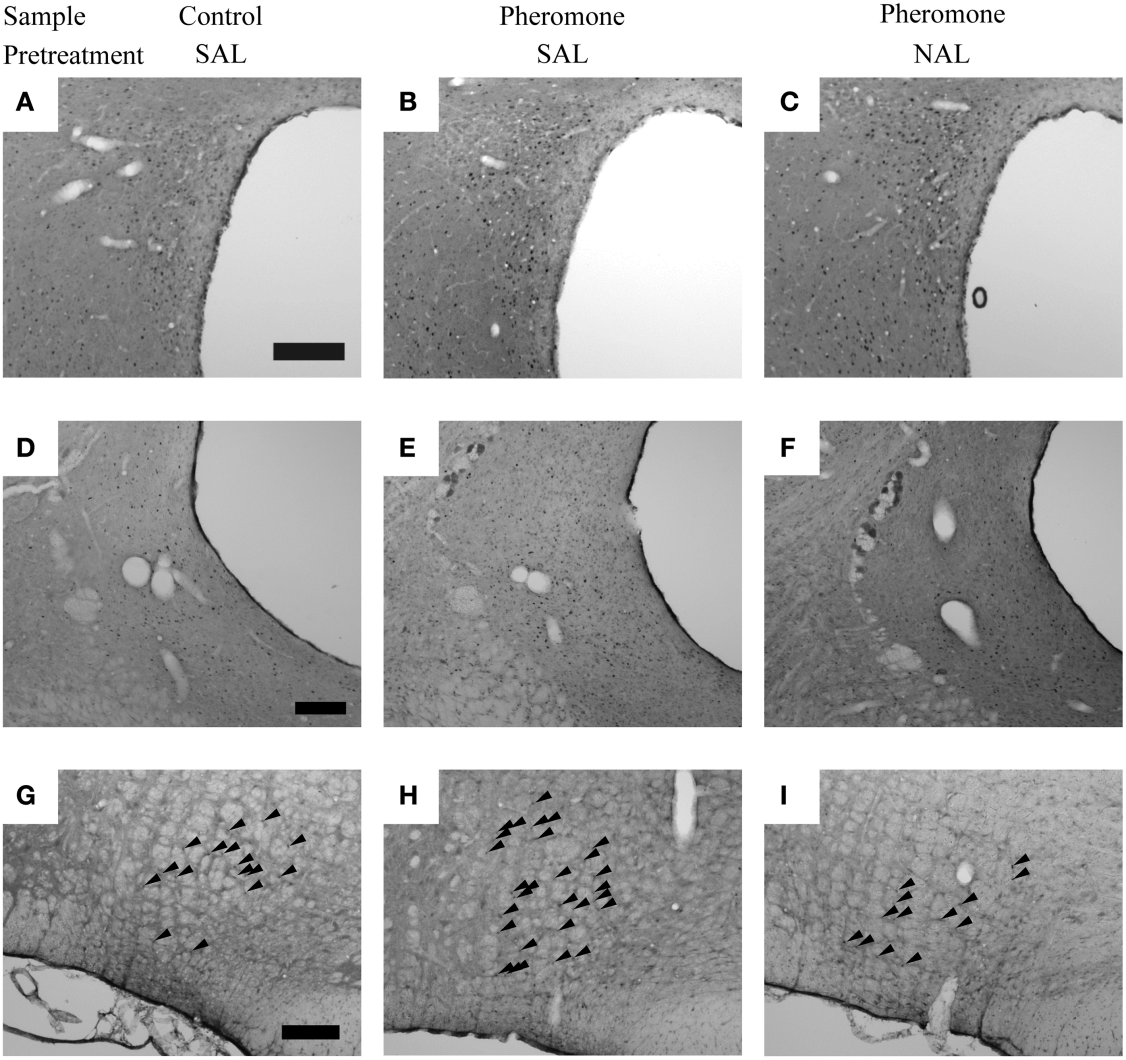
**The nuclei in which pretreatment with naloxone attenuated the pheromone-induced changes in Fos expression**. Photomicrographs showing Fos expression in the paraventricular nucleus of the hypothalamus **(A–C)**, ventrolateral periaqueductal gray **(D–F)**, and nucleus paragigantocellularis of rats **(G–I)** that were pretreated with either saline (SAL) or naloxone (40 mg·kg^−1^; NAL) and exposed to a control odor (Control) or an alarm pheromone (Pheromone). Arrowheads indicate Fos expression. The scale bar represents 200 μm.

*Post-hoc* tests revealed that these brain regions could be divided into the following 3 categories. The first category is composed of the mPVN (Figures 3A–C), vlPAG (Figures 3D–F), and nPGi (Figures 3G–I), in which pretreatment with naloxone blocked the pheromone-induced change in Fos expression. Compared with the Control-SAL group, the Pheromone-SAL group (*P* = 0.003), but not the Pheromone-NAL group (*P* = 0.795), showed decreased Fos expression in the mPVN. Fos expression in the vlPAG and nPGi in the Pheromone-SAL group (vlPAG, *P* < 0.001; nPGi, *P* < 0.001), but not the Pheromone-NAL group (vlPAG, *P* = 0.969; nPGi, *P* = 0.226) was increased compared with that in the Control-SAL group. The second category is composed of the BNSTam, BNSTal, BNSTp, pPVN, Arc, and dlPAG, in which pretreatment with naloxone did not block the pheromone-induced increase Fos expression. Both the Pheromone-SAL (BNSTam, *P* = 0.009; BNSTal, *P* = 0.012; BNSTp, *P* = 0.004; pPVN, *P* = 0.036; Arc, *P* = 0.001; dlPAG, *P* = 0.007) and Pheromone-NAL groups (BNSTam, *P* = 0.020; BNSTal, *P* < 0.001; BNSTp, *P* = 0.005; pPVN, *P* < 0.001; Arc, *P* < 0.001; dlPAG, *P* = 0.011) showed increased Fos expression when compared with the Control-SAL group in these regions. The last category is composed of the nucleus accumbens core and shell, ventromedial nucleus of hypothalamus, central amygdala, and dorsal raphe nucleus, in which pretreatment with naloxone increased Fos expression but the alarm pheromone did not. In these regions, the Pheromone-NAL group (nucleus accumbens core, *P* = 0.001; nucleus accumbens shell, *P* = 0.020; ventromedial nucleus of hypothalamus, *P* = 0.005; central amygdala, *P* < 0.001; dorsal raphe nucleus, *P* = 0.005), but not the Pheromone-SAL group (nucleus accumbens core, *P* = 0.470; nucleus accumbens shell, *P* = 0.436; ventromedial nucleus of hypothalamus, *P* = 0.568; central amygdala, *P* = 0.939; dorsal raphe nucleus, *P* = 0.813), showed increased Fos expression compared with the Control-SAL group.

*Post-hoc* tests confirmed a significant difference between the Pheromone-SAL and Pheromone-NAL groups in nuclei in the first (mPVN, *P* = 0.001; vlPAG, *P* < 0.001; nPGi, *P* < 0.001) and third categories (nucleus accumbens core, *P* = 0.006; nucleus accumbens shell, *P* = 0.003; ventromedial nucleus of hypothalamus, *P* = 0.020; central amygdala, *P* < 0.001; dorsal raphe nucleus, *P* = 0.009). Similarly, Fos expression was not different between the Pheromone-SAL and Pheromone-NAL groups in nuclei in the second category (BNSTam, *P* = 0.665; BNSTp, *P* = 0.787; pPVN, *P* = 0.090; Arc, *P* = 0.437; dlPAG, *P* = 0.774), except in the BNSTal, where the Pheromone-NAL group showed increased Fos expression compared with the Pheromone-SAL group (*P* = 0.034).

## Discussion

**Figure 4:**
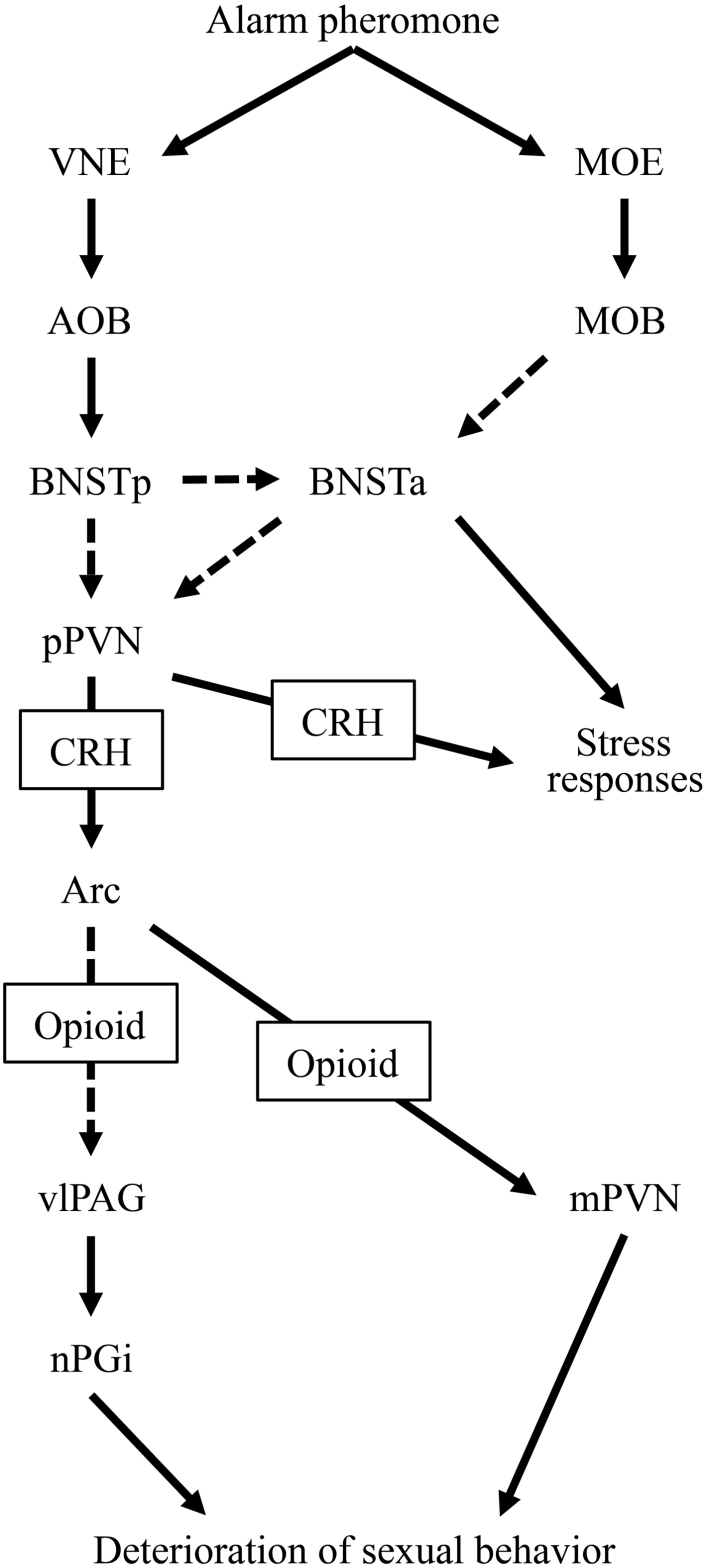
**A diagram of the hypothesized neural circuit inducing deterioration in sexual behavior by the alarm pheromone**. Solid arrows indicate direct projections while dotted arrows indicate indirect or unclear projections. Abbreviations: AOB, accessory olfactory bulb; Arc, arcuate nucleus; BNSTa, anterior division of the bed nucleus of the stria terminalis; BNSTp, posterior division of the bed nucleus of the stria terminalis; CRH, corticotropin-releasing hormone; MOB, main olfactory bulb; MOE, main olfactory epithelium; mPVN, magnocellular part of the paraventricular nucleus of hypothalamus; nPGi, nucleus paragigantocellularis; pPVN, parvocellular part of the paraventricular nucleus of hypothalamus; vlPAG, ventrolateral periaqueductal gray; VNE, vomeronasal epithelium.

In the present study, we attempted to clarify the neural mechanisms underlying the alarm pheromone-induced deterioration in sexual behavior in male rats and assessed how opioids were involved. We observed that the pheromone affected Fos expression in the BNSTam, BNSTal, BNSTp, mPVN, pPVN, Arc, dlPAG, vlPAG, and nPGi. In addition, pretreatment with naloxone blocked these effects in the mPVN, vlPAG, and nPGi. Based on these results, we have revised our hypothesis regarding the neural mechanisms underlying the alarm pheromone-induced deterioration in sexual behavior (Kobayashi et al., 2013a, b) as follows: when a male rat detects the alarm pheromone, the vomeronasal system, including the BNSTp, and the main olfactory system (Inagaki et al., 2014) receive information about the pheromone. Information from these 2 olfactory systems activates the pPVN, which subsequently activates opioidergic neurons in the Arc. The opioidergic neurons suppress the mPVN and indirectly activate the vlPAG-nPGi cluster. As a result, sexual behavior deteriorates (Figure 4).

In addition to the nuclei found in our previous study, and excluding the neural responses related to ejaculation, we found activation in the Arc, dlPAG, and vlPAG and suppression in the mPVN during deterioration in sexual behavior. Among these brain regions, the vlPAG and mPVN might be directly involved in the deterioration. It has been reported that lesioning the vlPAG reduces the number of mounts (Clark, 1975), suggesting that vlPAG activation deteriorates sexual behavior. The modulatory effects of the vlPAG might be exerted through the nPGi because lesioning the vlPAG (Clark, 1975) and nPGi (Yells et al., 1992) evokes the same pattern of deterioration. In addition, the vlPAG sends dense projections to the nPGi, which are activated during sexual behavior (Normandin and Murphy, 2008). Therefore, the vlPAG and nPGi might compose a cluster that modulates sexual behavior. Similarly, the mPVN might be involved in the deterioration; it has been reported that lesions in both the mPVN and pPVN, but not in the pPVN, increase the number of mounts and decrease the hit rate (Liu et al., 1997a). Therefore, it has been suggested that suppression of the mPVN deteriorates male sexual behavior. However, the mPVN might deteriorate sexual behavior in parallel with the vlPAG-nPGi cluster because, to the best of our knowledge, the mPVN and vlPAG-nPGi cluster are not anatomically connected. In contrast, activation of the dlPAG seems to be less important for the deterioration of sexual behavior. Anatomical evidence suggests that this region receives sensory information regarding sexual behavior from genital organs in the female (Klop et al., 2005). This information might also activate the dlPAG of the male in the present study. The Arc might be indirectly involved in the deterioration as discussed below.

Based on the present findings, we suggest that naloxone attenuated, rather than compensated for, the alarm pheromone-induced deterioration in sexual behavior. In the present and previous studies, rats that were pretreated with naloxone did not show a pheromone-induced deterioration in sexual behavior (Kobayashi et al., 2013b). One possible interpretation of this is that naloxone worked as an antagonist to the pheromone. Naloxone facilitates sexual behavior (McIntosh et al., 1980) and an opioid receptor antagonist increases Fos expression in nuclei that are not related to the nPGi, such as the nucleus accumbens core and shell, BNSTal, and central amygdala (Park and Carr, 1998; Carr et al., 1999). Therefore, an alternative interpretation could be that naloxone compensated the pheromone effects by facilitating sexual behavior through a separate neural mechanism. However, in the present study, we found that pretreatment with naloxone attenuated the pheromone-induced activation and suppression of the vlPAG-nPGi cluster and mPVN, respectively. Therefore, these results support the former interpretation.

Alarm pheromone-induced activation and suppression of the vlPAG-nPGi cluster and mPVN, respectively, were blocked by pretreatment with naloxone; therefore, these changes might be mediated by opioids. One possible source of opioids might be the Arc. Although its direct role in the deterioration of sexual behavior remains to be studied, the Arc contains abundant opioid peptides (Le Merrer et al., 2009), which are released by several stressors (Marinelli et al., 2004). In addition, the Arc sends direct projections to the mPVN (Sawchenko and Swanson, 1983); this suggests that opioid neurons in the Arc could directly suppress the mPVN. Indeed, an injection of opioids into the PVN inhibits male sexual behavior (Melis et al., 1999). The Arc could also affect the vlPAG-nPGi cluster because it sends projections to the medial preoptic area (MPOA) (Horvath et al., 1992). Electrical stimulation of the MPOA activates neurons in the vlPAG that project to the nPGi (Rizvi et al., 1996). Taken together, we propose that the Arc is the source of opioids that deteriorated the sexual behavior in the present study by suppressing the mPVN and activating the vlPAG-nPGi cluster.

In contrast to the suggested potential role of the MPOA, in our study, Fos expression in this region did not show any difference attributable to either the alarm pheromone or the pretreatment with naloxone. It is possible that the neural activation related to the expression of sexual behavior and/or intromissions obscured the alarm pheromone-induced difference in Fos expression. In this study, all the animals expressed 10 intromissions with equivalent latencies to mount, intromission, and tenth intromission, even when the alarm pheromone increased the number of mounts and decreased hit rate. It is known that the MPOA plays an important role in the expression of sexual behavior (Liu et al., 1997b). In addition, Fos expression in the MPOA is increased in subjects after 5 intromissions (Baum and Everitt, 1992). Therefore, high levels of Fos expression induced by the expression of sexual behavior and/or 10 intromissions might prevent us from observing any alarm pheromone-induced change of Fos expression in this region.

In the present study, we found that alarm pheromone-induced activation of the BNSTam, BNSTal, BNSTp, pPVN, and Arc was not blocked by pretreatment with naloxone. In the context of our hypothesized neural circuit that has been discussed above and in our previous study (Kobayashi et al., 2013a, b), these nuclei might be upstream of opioidergic neurons in the Arc. The alarm pheromone is perceived by the vomeronasal epithelium (Kiyokawa et al., 2007, 2013a), which transmits information to the accessory olfactory bulb, and subsequently, the BNSTp. This information is transmitted to the anterior BNST (BNSTa), composed of BNSTal and BNSTam, and the pPVN. It is currently unclear whether the information is transmitted from the BNSTp to the BNSTa and pPVN directly or indirectly. The BNSTa is only activated when it receives simultaneous information from the main olfactory system (Inagaki et al., 2014), which enhances the responsiveness of the pPVN to the stimuli (Inagaki et al., 2014). The activated pPVN sends CRHergic output to the Arc and induces opioid secretion. Therefore, pretreatment with naloxone may not affect the activity in these nuclei (Figure 4).

In summary, an alarm pheromone increased the number of mounts and decreased the hit rate in male rats. This was accompanied by an increase in Fos expression in the BNSTam, BNSTal, BNSTp, pPVN, Arc, dlPAG, vlPAG, and nPGi and a decrease in the mPVN. Naloxone attenuated the pheromone-induced deterioration in sexual behavior and modification of Fos expression in the mPVN, vlPAG, and nPGi. Based on these results, we suggest that the alarm pheromone activates opioidergic neurons in the Arc, which deteriorate male sexual behavior by activating the vlPAG-nPGi cluster and suppressing the mPVN.

### Conflict of interest statement

The authors declare that the research was conducted in the absence of any commercial or financial relationships that could be construed as a potential conflict of interest.
